# The Southampton-York Natural Scenes (SYNS) dataset: Statistics of surface attitude

**DOI:** 10.1038/srep35805

**Published:** 2016-10-26

**Authors:** Wendy J. Adams, James H. Elder, Erich W. Graf, Julian Leyland, Arthur J. Lugtigheid, Alexander Muryy

**Affiliations:** 1 Centre for Vision and Cognition, Psychology, University of Southampton, UK; 2 Centre for Vision Research, Department of Psychology, Department of Electrical Engineering & Computer Science, York University, Canada; 3 Geography and Environment, University of Southampton, UK

## Abstract

Recovering 3D scenes from 2D images is an under-constrained task; optimal estimation depends upon knowledge of the underlying scene statistics. Here we introduce the Southampton-York Natural Scenes dataset (SYNS: https://syns.soton.ac.uk), which provides comprehensive scene statistics useful for understanding biological vision and for improving machine vision systems. In order to capture the diversity of environments that humans encounter, scenes were surveyed at random locations within 25 indoor and outdoor categories. Each survey includes (i) spherical LiDAR range data (ii) high-dynamic range spherical imagery and (iii) a panorama of stereo image pairs. We envisage many uses for the dataset and present one example: an analysis of surface attitude statistics, conditioned on scene category and viewing elevation. Surface normals were estimated using a novel adaptive scale selection algorithm. Across categories, surface attitude below the horizon is dominated by the ground plane (0° tilt). Near the horizon, probability density is elevated at 90°/270° tilt due to vertical surfaces (trees, walls). Above the horizon, probability density is elevated near 0° slant due to overhead structure such as ceilings and leaf canopies. These structural regularities represent potentially useful prior assumptions for human and machine observers, and may predict human biases in perceived surface attitude.

A key function of the human visual system is to recover the 3D structure of the world from 2D images. This problem is highly complex and ill-posed - any single retinal image is consistent with an infinite set of 3D scenes varying in shape, reflectance and illumination. To perform well in natural environments, the visual system must exploit the joint statistics of natural scenes and the projected retinal images. Research on the connection between these statistics and visual processing can be divided into three broad categories^1^:

(i) *Image statistics*: Several studies have demonstrated that retinal and cortical encoding of image properties such as luminance, contrast and colour are adapted to the statistics of natural images to maximize coding efficiency^2^,^3^,^4^,^5^,^6^,^7^,^8^,^9^,^10^ and sparseness of neural response^11^. Biases in orientation perception have been related to statistical anisotropies in natural images^12^ and principles of perceptual organization^13^,^14^,^15^ have been found to quantitatively reflect the statistics of geometric relationships between local contour segments in natural images^16^,^17^,^18^,^19^. Moving stimuli are perceived to move slower at lower contrasts, and this can be accounted for quantitatively as a statistical prior for slower speeds^12^.

(ii) 3D *Scene statistics*: Given the inherent ambiguity of visual images, estimates of 3D scene properties should be biased toward those that are most common. For example, the surface depicted in Fig. 1a is usually perceived to have a depth configuration that is consistent with both illumination and viewpoint from above, rather than below (i.e., even numbered ridges are convex), in accordance with our experience of overhead lighting^2^,^20^,^21^, and the greater prevalence of visible surfaces whose normals point upward, rather than downward^22^,^23^. In Bayesian terms, these biases reflect prior probability distributions over scene variables, and the Bayesian framework provides a rigorous way to optimally combine prior information with current sensory data. By incorporating priors over scene properties such as surface orientation^24^, surface curvature^25^,^26^,^27^,^28^ and illumination^23^,^29^,^30^,^31^ this approach has been successful in explaining human biases in 3D visual perception. Similarly, priors over illumination, shape and reflectance have aided recent computer vision algorithms for single-view estimation of these variables^32^,^33^.

When inferred from perceptual data, these priors represent internal models of world properties, and fully understanding perceptual inference entails comparing these internal priors to objective priors estimated from scene statistics. In computer vision work^33^ these priors have been estimated from objective scene statistics, but often from a relatively small dataset and narrow range of scenes. In human vision work, these priors have been estimated from perceptual data but, with important exceptions^12^, these perceptual priors have generally not been compared to objective scene statistics. To estimate accurate and broadly applicable objective priors and validate measured internal priors, a diverse and comprehensive dataset of natural scene statistics is required.

(iii) *Image – 3D scene relationships:* Image features such as gradients and discontinuities in colour and texture provide information about scene structure, such as surface attitude, surface curvature and depth discontinuities. However, these image cues are typically co-determined by multiple scene variables. For example, local luminance variation in the image (e.g., Fig. 1) is generally co-determined by the shape and reflectance of the visible surface and the way in which it is illuminated. Similarly, a luminance discontinuity in the image might arise from an object boundary or a cast shadow. For this reason, the visual system must learn probabilistic image – scene relationships – likelihood functions in Bayesian terms.

Previous analyses of natural scenes have identified some novel image – scene relationships. For example, previous studies of 3D scene statistics have revealed that surfaces further from the observer tend to be darker^34^,^35^, which might explain analogous perceptual biases measured in the laboratory^36^,^37^,^38^. Other statistical studies have found that discontinuities are larger when the occluding contour is convex, and this relationship is mirrored by biases in human estimatation of depth^39^.

## Natural 3D Scenes Datasets

There have been a number of efforts in recent years to gather 3D scene statistics to inform human and computer vision research. Huang *et al*.^40^ collected 54 panoramic Light Detection and Range (LiDAR) range images in Rhode Island and Massachusetts forests in late summer, and performed some simple analyses of first- and second-order point statistics of the range data. Howe & Purves^41^ acquired registered LiDAR range and colour imagery for 25 natural scenes from the campus of Duke University and nearby Duke Forest and used these to analyse the statistics of projected lines. Potetz & Lee^34^ gathered over 100 outdoor scenes of registered LiDAR range and colour imagery under sunny summer conditions in western Pennsylvania and identified surprisingly strong linear correlations between range and luminance measurements. Su *et al*.^42^ acquired 12 scenes of registered LiDAR range and colour imagery from the campus of the University of Texas and the Texas State Capitol in Austin and studied the co-dependency between local range and range gradient and the response of Gabor filters to the registered colour imagery^21^.

In addition to LiDAR datasets, a number of recent datasets have employed low-cost structured-light range sensors such as Microsoft’s Kinect. For example, the NYU Depth Dataset V2^43^ provides registered range and colour imagery for 464 indoor scenes. These structured light datasets are very useful but qualitatively different from LiDAR datasets: they have much lower resolution, are much noisier, and typically restricted to indoor scenes.

While these prior efforts have all contributed insights into the statistical foundations of visual inference, there are a number of limitations that SYNS addresses:

1. Sampling. In prior work, sites were generally selected by convenience: scenes tend to be at or near university campuses, and data were collected in a single season. These constraints could result in low diversity and bias.
2. Stereo. Most prior datasets provide registered range and colour imagery, but do not include stereo pairs, which are important to understanding stereopsis in humans and for the development of optimal stereo algorithms for machine vision. (Stereo datasets such Middlebury^44^ provide stereo pairs, but are not designed to capture natural scene structure; most cover a small field of view and depict staged indoor scenes).
3. Resolution. Prior sensing technologies had lower resolution, both spatially and photometrically, than what can be achieved today.
4. Field of view. Most prior datasets provide images with a limited field of view, whereas good spherical imaging systems are now available.
5. Availability. Prior LiDAR datasets are generally not publicly available. This limits the impact these datasets have in the research community. (An exception is the 12-scene LIVE dataset^42^).

The remainder of this paper proceeds in two parts. In the first part, we describe the properties of the SYNS dataset and how it was constructed. In the second part we provide an example of the utility of the dataset by deriving ecological statistics for 3D surface attitude in natural scenes.

## The SYNS Dataset

### Sampling Strategy

**Figure 2:**
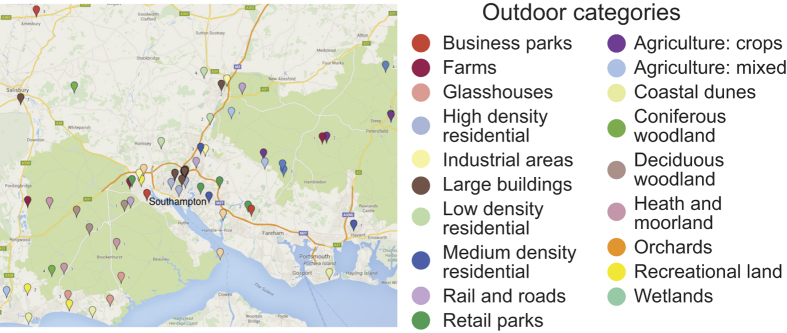
Sampled locations and land-use categories. Map data © 2016 Google.

Previous efforts to build natural scene datasets employed opportunistic sampling methods, selecting locations on or near a university campus, for example^21^,^41^,^45^,^46^. To achieve a broader and less biased sampling, we selected outdoor scenes from categories identified by the UKLand dataset (The GeoInformation Group; www.geoinformationgroup.co.uk), which provides high-resolution (from 50 m  × 50 m) land use classification, based on Ordnance Survey Open Data and aerial photography. We identified a 5,000 km^2^ circular region of interest (ROI) within 40 km of the University of Southampton campus in Hampshire, UK containing 23 of the 27 UK land use categories. Four of these 23 categories were excluded from our study due to their inaccessibility (these were mining and water-based categories), resulting in 19 categories for our study. Within each of these categories, three locations were selected randomly and uniformly within our 5,000 km^2^ ROI using Esri’s ArcGIS mapping platform (http://www.esri.com; Fig. 2), for a total of 57 outdoor sites. Of these 57 sites, 37 were sampled in the summer, 20 were sampled in the winter/early spring, and 19 (one for each scene category) were sampled twice, once in summer and once in winter/early spring, to allow for analysis of seasonal variations in scene statistics (future work). The result is a total of 76 outdoor scans.

Unbiased sampling of indoor categories is more challenging due to accessibility restrictions. We identified six indoor categories available within the university campus and within nearby private homes: (1) foyers, (2) classrooms, (3) offices, (4), living rooms (5) restaurants/cafés and (6) theatres/lecture halls. Where possible, locations were randomly selected within the available sites on campus (i.e. randomly sampling a building, latitude and longitude coordinates and a floor number within the building). Locations with limited accessibility (living rooms, theatres, cafes) were sampled according to availability. Two sites in each indoor category were sampled, yielding a total of 12 indoor scenes. Thus the SYNS dataset consists of 88 scenes in total (76 outdoor, 12 indoor).

### Modalities

At each sample location, three types of data (range data, HDR spherical imagery and stereo image pairs) were captured in sequence, from a common vantage point 165 cm above ground level to approximate human eye height (see Methods for full details of equipment, sampling process, calibration, co-registration and artefact removal).

### Range Data

**Figure 3:**
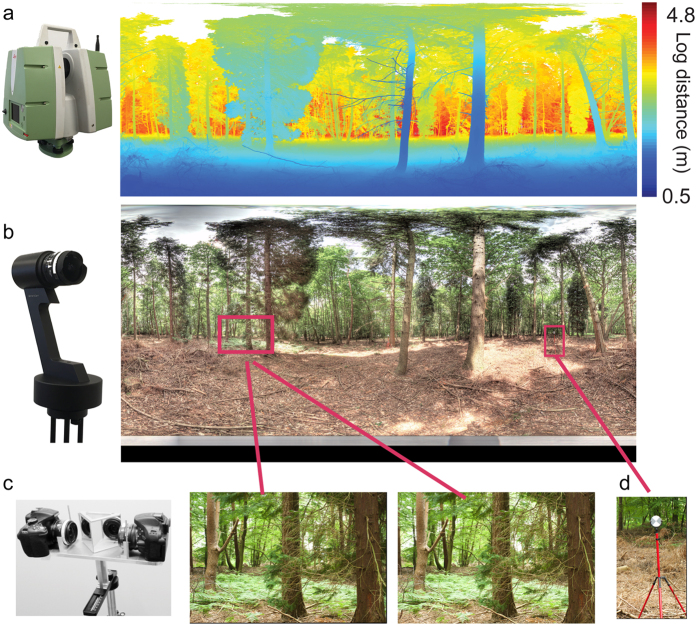
(**a**) LiDAR scanner with example range data (**b**) Spheron SpheroCam with example spherical image. (**c**) Stereo rig with example stereo pair, (**d**) High contrast target used for co-registration.

Range data were captured with a scanning LiDAR system (Leica ScanStation P20, Leica Geosystems Ltd., see Fig. 3a), which determines distances to scene points via the return time of laser light pulses (i.e., time-of-flight). Each LiDAR scan covers a 360° horizontal × 135° vertical field of view (FOV), essentially a spherical FOV that excludes the footprint of the LiDAR tripod, with a maximum range of 120 m. Angular resolution for this device is selectable: higher resolutions entail longer scan times. We chose an angular resolution of 2.2 arcmin, which results in a maximum theoretical scan size of 10,054 × 3,771 returns. (In practice, not all directions generate a return.) Example range data are shown in Fig. 3a. The scanning beam sweeps rapidly in the elevation direction, and rotates more slowly in the azimuthal direction. At our chosen resolution, one scan takes approximately 6.5 minutes. We captured two back-to-back scans per scene, allowing estimation of reliability and removal of large motion artefacts (e.g. moving people and cars – see Methods for details).

### High Dynamic Range Panoramas

High dynamic range spherical images were acquired using a Spheron SpheroCam HDR (www.spheron.com, Fig. 3b). This device captures a spherical (360° × 180°) colour image; a small area under the camera (0°–11.5° elevation) is occluded by the tripod but still recorded. The camera rotates around a vertical axis whilst capturing vertical image slices through a fish-eye lens. Within each scan, aperture size and central exposure time are manually selected and fixed. High dynamic range is achieved by capturing each image slice with multiple exposure times and sensor gains, up to 26 ‘stops’, where 1 stop is equivalent to doubling the exposure time.

At the settings we selected (26 stops, 4 arcmin spatial resolution in azimuth and elevation, resulting in a 5,400 × 2,700 pixel HDR image), each scan took 5–10 minutes, depending on the central exposure. We captured two back-to-back scans per scene, as well as one 2 minute, lower dynamic range (12 stop) scan to reduce illumination changes due to cloud motion during the scan.

### Stereoscopic Image Pairs

Stereo images were captured with a custom-built stereo rig employing two Nikon D5200 digital SLR cameras with AF Nikkor 35 mm f/2D lenses (Fig. 3c). 45° mirrors were used to achieve a stereo baseline (distance between the virtual nodal points) of 63 mm, matching the average human inter-pupillary distance^47^,^48^. The view vectors of the two cameras were parallel. Each camera had a relatively small (36.5° × 24.3°) FOV; to cover the full 360° horizontal panorama the rig was rotated around a vertical axis through the cyclopean point bisecting the two camera nodal points to capture 18 pairs of stereo images at 20° intervals. Stereo images are 6000 × 4000 pixels, corresponding to a spatial resolution of 0.36 arcmin.

### Alignment and Co-registration

Efforts were made to align the nodal points of the LiDAR and SpheroCam and the cyclopean point of the stereo rig as closely as possible. All devices were mounted on the same Leica surveying tripod, stabilized and levelled to within 8 arcmin. Sensors were mounted sequentially onto the tripod, using custom height adaptors. To enable accurate co-registration and to correct for residual offsets between the devices, three high-contrast targets (15cm diameter) were placed at roughly 120° azimuthal spacing in each scene (Fig. 3d).

During post-processing, targets were localised within each LiDAR scan to 0.1mm precision using Leica Cyclone software. Within the HDR images, target coordinates were estimated with 0.1 pixel (0.4arcmin) resolution using the ‘cornerfinder’ function provided by the contributed Camera Calibration MATLAB toolbox (www.vision.caltech.edu/bouguetj/calib_doc/). In order to align LiDAR and Spheron data for each scene, we first represented the 3D locations of each of the three targets in the coordinate frame of the LiDAR as vectors **l**_**i**_, *i* ∈ {1, 2, 3}, and the directions of these three targets in the coordinate frame of the Spheron as unit vectors 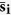, *i* ∈ {1, 2, 3}. We then estimated the 3D translation vector 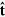 and rotation matrix 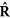 of the LiDAR frame relative to the Spheron frame by translating and rotating the LiDAR vectors **l**_**i**_ to minimize the sum of squared deviations between the transformed and normalized LiDAR vectors and the Spheron unit vectors 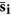:


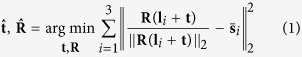


**Figure 4:**
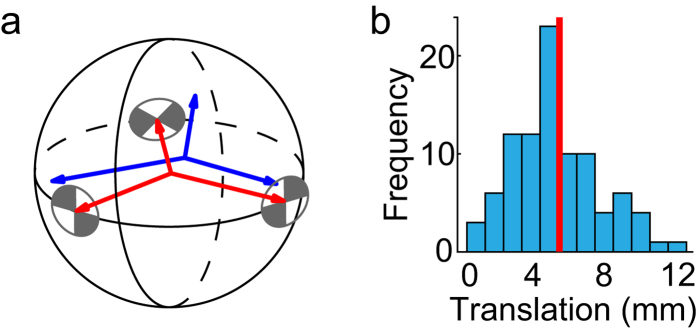
Co-registration of LiDAR and Spheron data. (**a**) The rigid transformation relating LiDAR and Spheron coordinate frames (red and blue) was estimated by aligning the projections of three calibration targets in the scene. (**b**) Magnitude of the estimated translational component of the rigid transformation over scenes. The red bar indicates the mean.

The mean length (across scenes) of the estimated translation vector 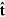 between the two sensors over all scenes was 5.8 mm (Fig. 4).

The stereo image pairs have yet to be co-registered with the HDR and LiDAR data; because the stereo cameras were horizontally offset by ±31.5 mm from the other sensors (to match human eye separation) there is no simple mapping from these images to the other two data types.

### Motion Artefact removal

Our LiDAR and Spheron sensors both require several minutes to complete a scan. This means that any environmental motion will lead to artefacts. We made an effort to minimize this problem by avoiding busy times with greater movement of people and cars, as well as windy or rainy conditions. However, to stay true to our random sampling protocol not all scene motion could be avoided.

**Figure 5:**
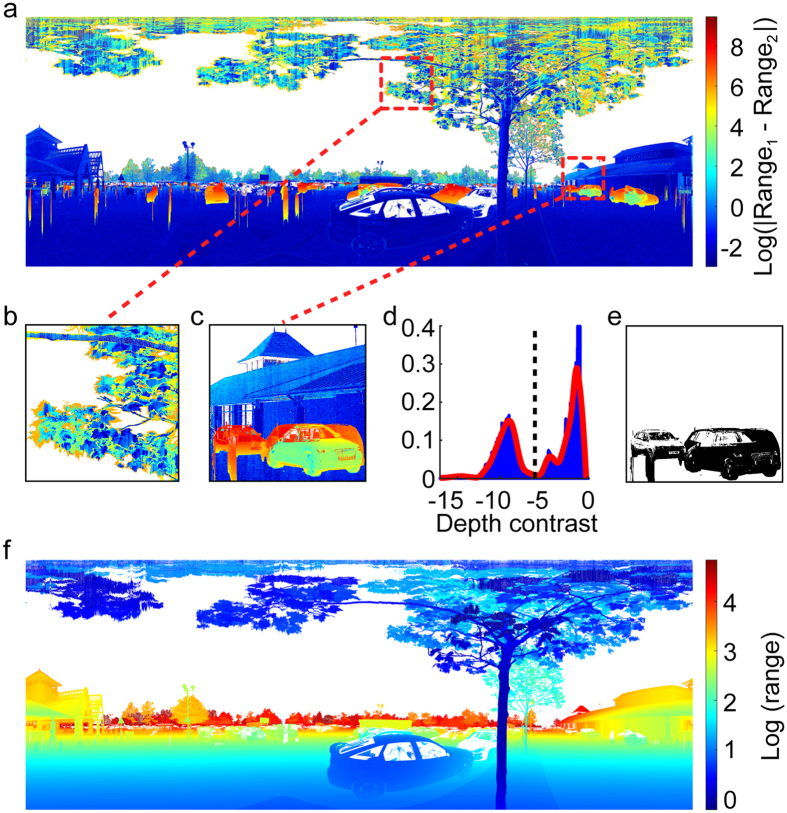
Motion artefacts. (**a**) Large absolute differences in range captured in the two scans indicate motion artefacts; laterally moving objects (e.g. people, cars) appear as thin vertical strips due to the rotational motion of the scanner. (**b**,**c**) Examples of foliage and vehicle/pedestrian motion. (**d**) Histogram of depth contrast for manually selected rectangular ROI with strong motion artefacts, with automatically selected threshold (dashed line). (**e**) Final mask defining the pixels to be assigned the maximum depth over the two scans. (**f**) Depth map with artefacts removed.

Some of these motion artefacts can be removed by comparing the two LiDAR scans captured at the scene: Fig. 5 illustrates the method for an example scene with a relatively large amount of environmental motion. In Fig. 5(a) we show the log of the absolute difference in range estimates between the two scans. Static regions of the scene appear as blue - the two scans agree. Foliage motion creates some smaller differences in estimated range between scans (Fig. 5b). However pedestrians and cars can create very large differences, as they may appear in one scan but not the other.

To remove artefacts associated with moving pedestrians and vehicles, bounding boxes around each were manually identified (Fig. 5c). The distribution of range contrast within each selected region was analysed to automatically identify a contrast threshold between the two major modes of the distribution (Fig. 5d). Values exceeding the threshold were classed as artefacts (Fig. 5e) and removed by replacing the range value with the larger of the two scan values (Fig. 5f). See Methods for additional technical details.

### Website

The SYNS dataset is public and freely available for download at https://syns.soton.ac.uk. For each scene we provide: (i) a LiDAR point cloud in PCD format (X-Y-Z-Return Intensity), with artefacts removed; (ii) Two HDR panoramas in OpenEXR and Radiance format aligned to the LiDAR data; (iii) 18 pairs of stereo-images in Nikon’s RAW (.NEF) and TIFF formats, forming a panorama centred on the horizon.

### Analysis of Surface Attitude

It is our hope that the SYNS dataset will contribute to many future insights into the statistical basis of visual perception. Here we initiate these analyses with a study of the distribution of surface attitudes revealed by our range data.

**Figure 6:**
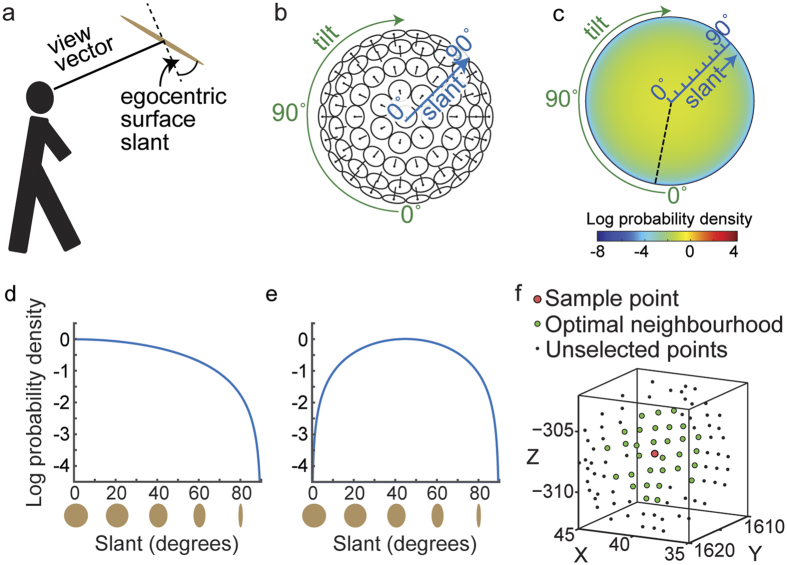
Coding of surface attitude and predictions. (**a**) Egocentric attitude is measured relative to the view vector. (**b**) This figure should be seen as a **concave** hemisphere, with pins pointing in the surface normal direction. (**c**) A maximum entropy world generates a distribution of observed surface attitudes that peaks at fronto-parallel. (**d**) For fixed tilt (e.g., dashed line in (**c**)), probability decreases with surface slant, following a cosine law, as shown by the foreshortening of the surface patches below and noted previously^67^. (**e**) When collapsed across tilt, probability peaks at 45°. (**f**) Selecting a neighbourhood to estimate surface attitude.

We consider surface attitude in egocentric coordinates, i.e., relative to the view vector. The view vector (egocentric z-axis) connects the sensor to the surface, as shown in Fig. 6a. The egocentric x and y axes lie in the image plane: the x-axis is always horizontal (in world coordinates) and the egocentric y-axis is orthogonal to the x- and z- axes, with positive y including an upward component. Since we positioned the nodal point of our LiDAR sensor at human eye height, the distribution of surface attitudes relative to the LiDAR view vector provides a good approximation to the distribution that would be experienced by a human observer in our scenes.

Egocentric surface attitude is typically expressed in terms of slant and tilt (Fig. 6a,b). Slant is the angle between the surface normal and the view vector: a frontoparallel surface has a slant of 0°, while a surface seen edge-on has a slant of 90°. Tilt is the angle of the projection of the surface normal in the image plane. We code this angle clockwise relative to the egocentric vertical axis in the image plane, so that a floor surface is assigned a tilt of 0°, a ceiling surface 180°, and left/right wall surfaces are assigned tilts of 90°/270°. Note that Fig. 6b maps slant and tilt onto a **concave** hemisphere – ceiling, ground, left and right wall planes lie at the top, bottom, left and right of the figure, respectively.

Previous work suggests that human observers tend to underestimate surface slant: surfaces are seen as closer to fronto-parallel than they really are^5^,^33^,^36^,^49^,^50^,^51^. This may be due in part to cue conflicts from fronto-parallel display screens^27^, but it may also be due to an adaptive bias learnt from the environment, if larger slants are encountered less frequently. To understand why such a bias might be present, consider the distribution of observable surface normals, **n**, in a scene (i.e., normals with a component in the direction of the observer), which can be represented as a probability density *p*_*N*_(**n**) on the unit hemisphere. In a maximum entropy random world, this density would be uniform and independent of viewing direction: 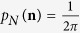.

We can represent these surface normals in egocentric spherical slant/tilt coordinates (*ϕ*, *θ*) where *ϕ* ∈ [0, *π*/2], *θ* ∈ [0, 2*π*) (Fig. 6a,b). In these coordinates, the probability density can be expressed as 
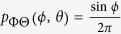
. (The factor of sin *ϕ* results from the fact that the surface patch delineated by a differential excursion in slant *ϕ* and tilt *θ* gets larger with increasing slant: *dA* = sin *ϕdϕdθ*).

This density specifies the probability of occurrence of a particular surface attitude (*ϕ*, *θ*). However, the probability of actually observing this attitude is also proportional to the area of the image projection of a surface patch with this attitude, and this projected area *A*′ declines with slant according to a cosine law: *A*′ ∝ cos *ϕ*. (This foreshortening effect is illustrated below the x-axis of Fig. 6d). The net result is that the probability density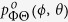 of *observed* surface attitude is given by


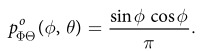


To visualize the probability density of surface attitudes in a 2D plot, we need to map the hemisphere of possible surface attitudes to the (*x*, *y*) plane. It is obviously desirable that a uniform density on the hemisphere appears as a uniform density in the plane. To achieve this, our mapping must be area-preserving. Many such mappings are possible; here we select one that provides an intuitive and natural visualization of surface attitude as a function of slant and tilt.

In particular, we define polar coordinates (*ϕ*′, *θ*′) in the (*x*, *y*) plane such that *ϕ*′ codes distance from the origin and *θ*′codes clockwise rotation from the −*y* axis:


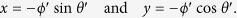


To produce an intuitive visualization we set the polar angle in the plane to equal the tilt angle: *θ*′ = *θ*, and set the radial coordinate in the plane to be a monotonic function of the slant: *ϕ*′ = *f*(*ϕ*).

To be area-preserving, we require that a differential element *dA*′ in the plane equal a differential element *dA* on the hemisphere:


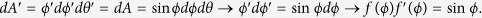


This equation is satisfied by the function 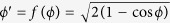. Note that the resulting radial coordinate *ϕ*′ ≃ *ϕ* for small *ϕ* but is slightly compressive, declining to 90% of the value of *ϕ* at *ϕ* = *π*/2 (90°).

This mapping from slant-tilt coordinates (*ϕ*, *θ*) to the (*x*, *y*) plane induces a transformation on the density:


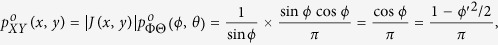


where *J*(*x*, *y*) is the Jacobian of (*ϕ*, *θ*) with respect to (*x*, *y*).

This density is shown in Fig. 6c. Note that the foreshortening of more slanted surfaces on projection to the image renders them less likely to be observed. As a result, this density attains its maximum at *ϕ* = 0 (fronto-parallel), decreasing monotonically to zero at *ϕ* = *π*/2 (90°, maximal slant). One implication of this is that if tilt is somehow known by the observer (e.g., fixed in an experiment), then the probability density falls as a cosine function of slant *ϕ* (Fig. 6d).

If tilt is not known, the one-dimensional probability density 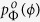 over slant *ϕ*, obtained from 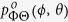 by marginalizing over tilt *θ*, is quite different: 
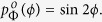
 This density is depicted in Fig. 6e. Note that it peaks at an intermediate slant of *ϕ* = *π*/4 (45°), falling to zero at *ϕ* = 0 (frontoparallel) and *ϕ* = *π*/2 (90°, maximal slant).

While this analysis provides a baseline for comparison, this maximum entropy model is unlikely to be a good approximation to our true visual world, where the ground plane and a preponderance of horizontal and vertical surfaces introduce statistical inhomogeneities, and so it remains to be seen whether the statistical distribution of surface attitude in the real world can explain perceptual biases measured in the laboratory.

### Surface Attitude Estimation

We used the SYNS LiDAR data to estimate a local planar approximation to the visible surface at every point in the 3D point cloud for each of our scenes. We then computed the egocentric slant and tilt parameters for each of these planes and averaged over scenes to estimate the probability density over egocentric surface attitudes.

The local planar surface model at a 3D point **X** will be based upon some number *k* of 3D points in the local neighbourhood of **X** (Fig. 6f). Given this neighbourhood, we approximate the position of the surface as the centroid and the surface normal as the smallest eigenvector of the spatial covariance of the 3D points^52^. A question immediately arises: what is *k*? Since three points define a plane, we clearly require *k* ≥ 3, however there is no clear upper bound. Given noise in the range data, using too small a neighbourhood will lead to noisy surface estimates. Large neighbourhoods, on the other hand, run the risk of over-smoothing potentially important smaller scale details in the 3D surfaces we are trying to estimate.

This is a classic scale selection problem that has seen considerable attention in both the computer vision^53^,^54^,^55^,^56^ and biological vision^57^ communities. Our approach is inspired by the minimum reliable scale principle introduced by Elder & Zucker for edge detection^55^. (This is also related to what Morel and colleagues call the Helmholtz principle^58^). Detection of an edge at a given image location is typically based on a logical decision function of one or more linear filters (e.g., Gaussian derivatives). The problem of selecting the scale of these filters typically involves a tradeoff between two sources of error: small scales result in lower signal-to-noise ratio, large scales increase the risk that filter responses will be contaminated by neighbouring structure in the image^59^.

While this latter risk is difficult to model, the variation of signal-to-noise with scale is more tractable^59^. Elder & Zucker modeled the noise as zero-mean Gaussian, independent and identically distributed (IID). Given known or estimated noise characteristics, a response threshold can be specified that will limit the probability of false detections (Type I errors) to a desired level (p-value). Importantly, this threshold declines as filter scale increases and the signal-to-noise ratio of the filter responses improves. Under the minimum reliable scale principle, the smallest scale is selected that exceeds threshold. If such a scale exists, the null hypothesis (no edge present) is rejected and an edge is detected.

The approach we take here is analogous. In the present work, an estimate of surface attitude is made at every 3D point in a point cloud, with a null hypothesis that the surface is locally planar. Whereas edge detection performance depends on filter scale, the accuracy of surface attitude estimation depends upon the size of the neighbourhood to which the planar model is fit. Small neighbourhoods yield noisy estimates, whereas larger neighbourhoods increase the risk of contamination from neighbouring structure (smoothing) and deviations from planarity. Since these latter effects are difficult to model, we balance this tradeoff by selecting the smallest neighbourhood that rejects the null hypothesis of planarity.

A deviation from Elder & Zucker’s approach is necessitated by a correlation in noise we observe in our LiDAR data, which breaks the IID assumption. To deal with this, we directly measure the statistics of the residual of the planar fit as a function of neighbourhood size, using point clouds returned from known planar surfaces. These statistics are used to determine the threshold for rejection of the null hypothesis.

Specifically, for each point in the scene, we fit a local plane for neighbourhoods increasing from *k* = 7 and select the first neighbourhood for which the hypothesis is rejected, up to a maximum neighbourhood size of *k* = 300 points. Technical details of the method, and comparison with an alternative unsupervised method, will be published in a companion paper.

**Figure 7:**
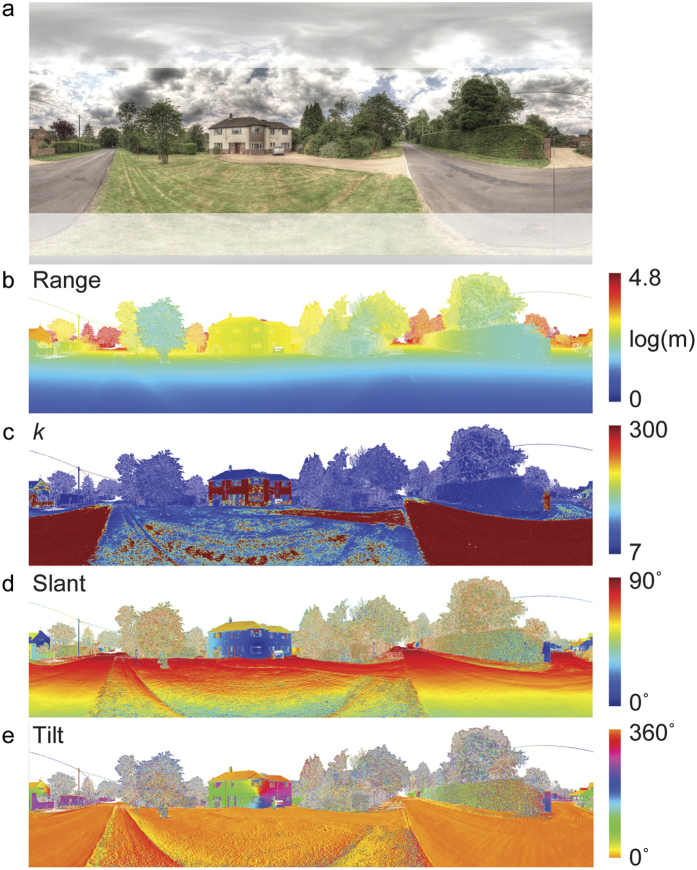
Surface attitude estimation for an example scene. (**a**) Spheron image; masked regions correspond to sky regions that do not generate LiDAR returns, and the low elevation region not captured by LiDAR. (**b**) Range data. (**c**) Optimal neighbourhood size. (**d**) Egocentric slant. (**e**) Egocentric tilt.

Figure 7 shows range data for an example scene, with the optimal neighbourhood size and estimated slant and tilt from our analyses. Our adaptive scale selection algorithm automatically selects small neighbourhoods where the scene structure is highly non-planar, such as grass and foliage, and adapts to large neighbourhoods where surfaces are relatively flat, such as the road surfaces and walls of the house.

## Results

We report surface attitude statistics for 69 scenes (all from unique sites). While the 57 outdoor scenes were distributed over 19 land-use categories, for this analysis we divided them into two categories: ‘outdoor natural’, or ‘outdoor built’ according to whether they contain predominantly built or predominantly natural structure. Outdoor scenes were labelled by 7 observers who viewed the HDR panoramas under Mollweide projection^60^ (under which relative area is preserved). The categories were assigned on the basis of majority vote resulting in 28 ‘natural’ scenes and 29 ‘built’ scenes. (All 7 observers were in agreement for 45 scenes; 5 or 6 observers were in agreement for 10 of the remaining 12 scenes).

**Figure 8:**
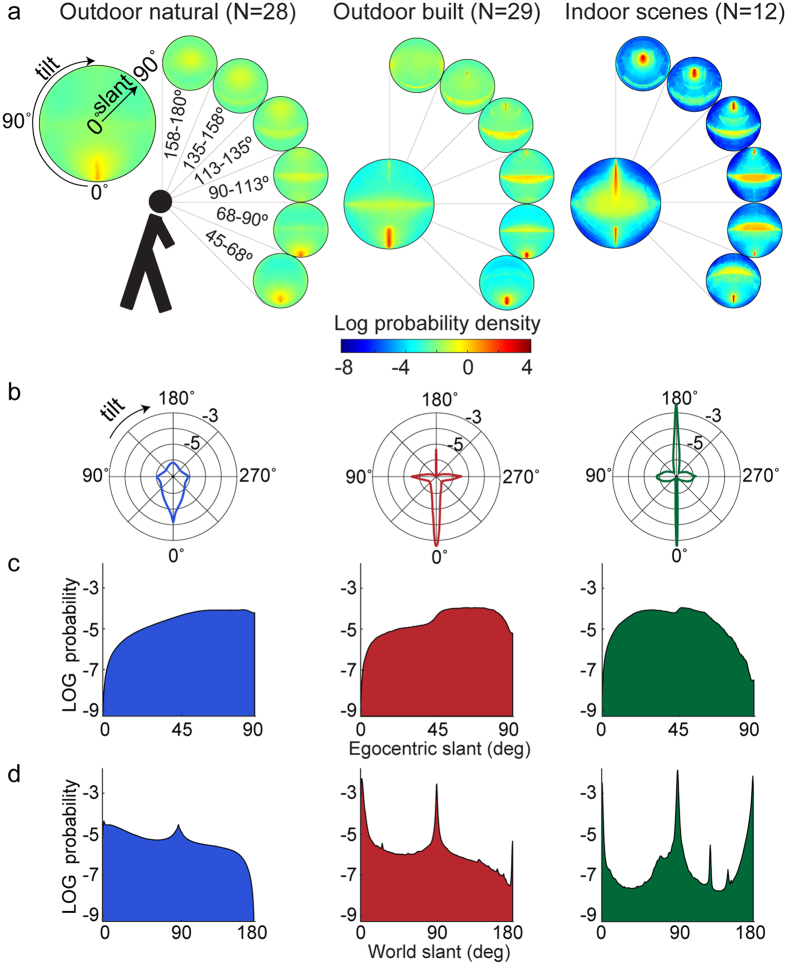
(**a**) Egocentric surface attitude for natural, built and indoor scenes. Each larger plot gives log(probability density) over slant and tilt, collapsed across elevation. Smaller plots give log(probability density), conditioned on a particular range of elevations. (**b**) Distribution of egocentric surface tilt, collapsed across slant, for the three categories, the radial axis gives log probability. (**c**) Distribution of egocentric slant, collapsed across tilt. (**d**) Distribution of slant (collapsed across tilt) in world coordinates, i.e. relative to gravity. Zero slant corresponds to horizontal surfaces viewed from above, 180° corresponds to horizontal surfaces viewed from below.

Figure 8a shows probability densities for these two outdoor categories and indoor scenes, and also breaks these down according to the elevation of the view vector. Several qualitative deviations from the random world hypothesis (Fig. 6c) are immediately apparent:

1. Horizontal surfaces. Figure 8a reveals a strong mode corresponding to horizontal surfaces seen from above (0° tilt) across all three categories, due in large part to the ground plane (note the orange regions in the lower regions of the scenes shown in Fig. 7e, and Supplementary Figs S1–S3). There is also a mode for all three categories at 180° tilt, corresponding to horizontal surfaces seen from below. For indoor scenes this mode is strong and well defined and arises predominantly from ceilings (as indicated by the blue regions in the upper part of Supplementary Fig. S1c). For outdoor scenes this mode is much weaker and may arise in part from overhead foliage, in addition to vertical surfaces (e.g. tree trunks) below the horizon (see Supplementary Fig. S3).
2. Vertical surfaces. There is a ridge of density around 90°/270° tilt corresponding to vertical surfaces in the scene. While present in all three categories, this ridge is strongest for indoor scenes and weakest for outdoor natural scenes, as might be expected. (The left-right symmetry of all density plots indicates that, as expected, leftward and rightward slanted surfaces are equally probable).
3. Frontoparallel surfaces. While the random world hypothesis predicts a symmetric peak in density at 0° slant (frontoparallel), we find that the situation is more complex for all three categories. For built and indoor scenes, density is high at frontparallel, but this is part of the extended ridge along the 90°/270° tilt axis corresponding to vertical surfaces in the scene, and no peak at frontoparallel is apparent for natural scenes.
4. Oblique surfaces. Indoor scenes include a much higher density of lower slant surfaces (up to around 40°) whose normals do not lie in either the horizontal or vertical meridian (as shown by the yellow region in the centre of the density plot). This is consistent with close vertical surfaces (walls) with a large vertical extent in the image (see Supplementary Fig. S1). In outdoor scenes, vertical surfaces are most common around the horizon, where they generate tilts near 90°/270° in egocentric coordinates.
5. Categorical diversity. In addition to the differences noted above, qualitatively the distributions differ dramatically across the three scene categories.
6. Elevation. Variation in the egocentric distribution of surface attitudes also varies profoundly according to the elevation of the view vector. When gaze is directed downward (45°–68° elevation) in outdoor scenes, surfaces are predominantly horizontal and viewed from above (e.g., the ground plane), while for indoor scenes there is also a ridge of density for higher tilts, arising predominantly from the nearby wall surfaces (see Supplementary Fig. S1). As gaze rises to the horizon we see the emerging dominance of vertical surfaces reflected in a ridge of density ranging around 90°/270° tilt. As gaze rises further above the horizon we see evidence for a mode near 180° tilt, i.e., horizontal surfaces seen from below, most pronounced for indoor scenes, consistent with ceiling planes.

Figure 8b–d further explore these statistics, collapsed over elevation angle. Figure 8b plots the probability density over egocentric tilt, collapsing over slant. Clear peaks at the four canonical tilt directions are seen for all categories, corresponding to horizontal and vertical surfaces in the scene. While the peak at 0° tilt (ground plane) is strong for all categories, we note that the other peaks are less pronounced for natural scenes. We also note that the peak at 180° (ceiling plane) is most pronounced for indoor scenes.

Figure 8c plots the density over slant, collapsing over tilt, and can be compared to the maximum entropy prediction in Fig. 6e. For natural scenes, density increases monotonically with slant, peaking near 90°. We believe this is due to the dominance of the ground plane and the increasing proportion of the view sphere that is sampled as elevation angle increases from 45° toward the horizon, where the ground plane approaches 90° slant. For built outdoor scenes, density peaks at an intermediate slant. We believe this is primarily due to the greater contribution of vertical surfaces, whose foreshortening creates a bias toward frontoparallel (note the large regions corresponding to small egocentric slants on the buildings in Supplementary Fig. S2). The influence of vertical surfaces is greater still for indoor scenes, where the most probable slant is reduced further, and the probability distribution over slant is closer to the maximum entropy prediction.

A sharp increase in density can be observed near 45° slant for both built and indoor scenes. Most likely, this is because we analyze elevations above 45° only (lower elevations are occluded by the tripod); the ground plane never generates an egocentric slant less than 45° as clearly seen at the lower edge of Fig. 7d).

Ego-centric slant and tilt are a function not only of the viewer’s (sensor’s) height above the ground, but the direction of the view vector, i.e., the vector connecting the optical centre to the surface point being analysed. Note that this view dependency will result in systematic deviations from the distribution of surface attitudes expressed in allocentric (world) coordinates. For example, in world coordinates, the slant of a horizontal ground plane remains the same (relative to gravity) over its entire extent. However, in ego-centric coordinates, the slant is greater for points on the ground plane that are more distant from the observer, as the view vector becomes more oblique to the ground plane.

To illustrate the full impact of view dependency, Fig. 8d shows the distribution of surface attitude as a function of world slant, coded as 0° for ground surfaces, increasing to 90° for vertical surfaces and on to 180° for ceiling surfaces. These plots highlight the strong peaks in density at the canonical directions corresponding to horizontal and vertical surfaces. A peak near 0° slant corresponding to ground surfaces is apparent for all categories, although weaker for natural environments. A peak near 90° corresponding to vertical surfaces is also apparent for all categories. Note that in egocentric coordinates these vertical surfaces generate a broad distribution of slants, with tilt concentrated near the 90°–270° axis. Finally, a peak near 180° corresponding to ceiling surfaces is apparent for built and indoor scenes. These deviations from the maximum entropy model generate the structured regularities apparent in the surface statistics shown in Fig. 8.

## Discussion

### Bias to Smaller Slants

It is clear that the statistics of egocentric surface attitude deviate substantially from the random world hypothesis, in ways that make sense given the preponderance of horizontal and vertical surfaces around us. But we can still ask whether our data are consistent with the underestimation of slant (bias toward fronto-parallel) that has previously been observed in the laboratory. To answer this question, it makes sense to consider density for the indoor condition, when gaze elevation is near horizontal, approximating the typical conditions for a psychophysical laboratory experiment. Under these conditions there is indeed a strong mode near frontoparallel (Fig. 8a).

Future studies can probe the relationship between natural scene statistics and human perception of surface attitude in more detail. For example, the mode near zero slant (for indoor scenes, gaze horizontal, as identified above) is much sharper along the meridional (0°–180° tilt) axis than along the azimuthal (90°–270° tilt) axis. This makes a strong prediction: if observers internalise these statistics, their prior for small slants should be stronger in a context where surfaces vary along the 0°–180° tilt axis than a context in which surfaces vary along the 90°–270° tilt axis. Prior studies^5^,^33^,^36^,^49^,^50^,^51^ demonstrating bias toward frontoparallel have varied surfaces either along the meridional or the azimuthal axis, but not both, and since methods vary widely between studies it is difficult to compare them directly. This prediction thus remains to be tested.

### Dependence on Scene Category and Elevation

The strong dependence of the distribution of surface attitudes on the category of scene (outdoor natural, outdoor built, indoor) and elevation generates the testable prediction that human observers may condition their estimates on these variables, and that machine vision systems should as well. For example, we might predict a bias toward zero tilt (related to the influence of the ground plane) when observers are looking downward, but not upward. Note that conditioning attitude judgements on elevation requires the observer to estimate the elevation angle of a point on the retina, which also depends upon gaze direction in world coordinates. Previous work has indicated that human observers do indeed have a representation of eye gaze. For example, after prism adaptation, observers’ slant judgments changed in accordance with the altered sense of eye position^61^.

Oliva and Torralba^62^ have argued that scene recognition may be based upon a set of holistic features, specifically: naturalness, openness, roughness, expansion and ruggedness, and that these dimensions can be reliably estimated from image content. In their work, these holistic features were described qualitatively. By providing registered 3D data with high-resolution imagery, the SYNS dataset affords the opportunity to explore quantitative definitions of these holistic features, and to assess their discriminative power in distinguishing semantic scene categories.

### Relationship to Prior Work

Yang and Purves^63^ have analysed the distribution of surface attitude for 3D point clouds extending ±40° above and below the horizon, derived from 23 forest scenes (see their Figs 5 and 6). They reported that surfaces “are most often slanted about horizontal or vertical axes” and later that surfaces are “more often slanted about the horizontal axis”. (These are the horizontal and vertical axes of a local virtual camera frame.) Unfortunately, a more detailed comparison with our results is not possible due to differences in the way that surface attitude was coded. In our analysis, we coded the surface attitude at a 3D point relative to the view vector, i.e., the ray passing through the optical centre and the 3D point. In contrast, Yang and Purves first divided their point cloud data into virtual 75° × 75° ‘images’ and then coded the surface attitude relative to the coordinate frame of each image.

## Conclusions

We have introduced the Southampton-York natural scenes (SYNS) dataset. The co-registered LiDAR and HDR imagery of SYNS and panorama of stereo pairs will benefit academic and industrial researchers interested in human and computer vision. We provide one example of the utility of the SYNS dataset: a quantitative analysis of surface attitude in real-world scenes. Multimodal probability densities reflect strong dominance of horizontal and vertical surfaces, and dependence on scene category and elevation suggests that conditioning on these variables will improve accuracy of surface estimation. We are currently quantifying psychophysical estimation of surface attitude^64^ to assess concordance with the statistics reported here and have been employing the Spheron HDR spherical images for light field modelling in 3D rendering^65^ and light adaptation models of the human retina^37^. We expect the SYNS dataset to be useful for a great diversity of future projects by laboratories around the world.

## Materials and Methods

### Scene Sampling

Within each of the 19 land-use categories considered in this study, spatial locations were selected randomly and uniformly within our 5,000 km^2^ ROI using Esri’s ArcGIS mapping platform (http://www.esri.com). Each randomly selected site was located using a high-precision (<1 m) GPS device. If the selected site was less than 1m from a large object such as a wall, the sensor was moved to increase this distance to at least 1 m. If the selected site was not accessible (e.g., permission could not be obtained), the closest accessible point was found within the same scene category. If no nearby accessible points were available, a new random sample was drawn from the category. After levelling the tripod and positioning the three co-registration targets, the devices were sequentially mounted to capture the three data types (LiDAR, SpheroCam, stereo rig). During a scan, all equipment was removed from the field of view. The entire process including set up and capture took roughly two hours per scene.

## LiDAR Range Data

### Technical Specifications

The Leica ScanStation P20 has nominal angular accuracy (azimuth and elevation) of 8 arcsec and range accuracy of 0.4 mm to 9 mm, depending on distance, surface albedo, and surface attitude. Our own measurements with known planar surfaces indicate RMS error of roughly 1 mm in the 2.5 m–50 m range. The scanner has a high-precision electronic level and built-in compensator, which automatically levels the device and corrects for small movements during a scan due to vibration.

### Procedure

A low resolution (17.4 arcmin) preview scan was captured first to determine the optimal focus distance for the spherical and stereo imagery (see below). We then captured two high-resolution scans spanning 362.25° in the azimuth direction (i.e., including a 2.25° overlap). The scans were cropped to 360° before aligning the two scans to enable motion artefact removal. Alignment was achieved to the nearest sample (8 arcsec) by maximizing the cross-correlation of the range data.

### Artefact Removal

The two range scans were used to identify and remove artefacts due to large objects moving across the scene (e.g. people, cars), as follows:

1. Bounding box. Artefacts were identified visually from the difference in log range between the two scans (Fig. 5a), and rectangular boxes bounding each artefact were selected manually (Fig. 5c).

2. Pixel segmentation. Within each bounding box, the range contrast of each pixel was identified:


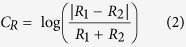


where *R*_1_ and *R*_2_ are the range estimates from the two scans. Smoothed histograms of range contrast within each bounding box reveal a bimodal distribution (Fig. 5d): small contrasts for pixels falling on the background and large contrasts for pixels falling on the moving object in one of the scans. Histogram smoothing was performed using the ‘smooth’ function from MATLAB, with the quadratic fit option. Segmentation was achieved by identifying the minimum of the smoothed histogram lying between the two largest modes and employing the corresponding range contrast as a threshold: only pixels with range contrast greater than this threshold were classified as artefact (Fig. 5e).

3. Range correction. For pixels classed as artefact, the smaller of the two range estimates was replaced with the larger estimate (Fig. 5f).

Other artefacts were also removed. Since low intensity returns tend to be noisy, all points with intensity <0.005, where maximum intensity is 1 were removed (~0.2% of points; these appear as [0, 0, 0, 0] in the dataset). In addition, highly reflective or specular surfaces (e.g. polished cars, glass, wet surfaces, traffic signs) and semi-transparent surfaces (e.g. water) can produce spurious returns. Where clearly identifiable, such points were removed manually using the Leica Cyclone software.

## HDR Spherical Imagery

### Technical Specifications

The SpheroCam has a Nikkor 16 mm fish-eye lens, allowing it to capture a 180° elevation FOV. During a scan, the camera rotates around its vertical axis, continuously capturing data at exposures that vary around a central (manually selected) exposure time; for indoor categories the mean central exposure was 1/15 sec, for outdoor scenes 1/50 sec. The camera’s sensor consists of 3 vertical charge-coupled device strips for red, green and blue channels. The resultant vertical image strips are automatically stitched into a 180° × 360° (elevation × azimuth) spherical image.

### Procedure

After completing the LiDAR measurements, the SpheroCam was mounted onto the tripod with a tribrach mount and re-levelled using the tribrach’s built-in bubble level. The (temperature-dependent) black point was determined using a black opaque lens cap supplied by Spheron. We employed Spheron’s ‘Cloudy Sky’ white balance preset for all scenes.

The focus setting was chosen for each scene individually. Using the range distribution from the low resolution LiDAR scan (spatial resolution = 17 arcmin), we calculated the resultant image blur circle^66^ for a range of focal distances at every point in the scan. The optimal focus setting was defined as that which minimised the mean focal blur circle diameter.

One or more low-resolution calibration scans were taken to identify the optimal exposure and aperture settings that centred the median luminance within the dynamic range of the camera. When possible, the aperture size was minimised to maximise the depth of field, subject to the constraint that the exposure setting remain less than 1/15 sec for outdoor measurements, to avoid very long scan times and increased effects of environmental motion and illumination change. When possible, scenes were captured during stable illumination conditions. For indoor scenes, the maximum central exposure was ¼ sec.

Illumination changes could not be avoided entirely. Supplementary Fig. S2 shows an example outdoor scene in which the sun’s occlusion varied due to cloud motion during capture – brighter vertical sections can be seen in the image as a result. Supplementary Fig. S2 also shows examples of motion artefacts that are present in some HDR images: two moving vehicles have been horizontally compressed in the image and are clearly visible as vertical strips.

## Stereo Panorama

### Technical Specifications

Two Nikon D5200 digital SLR cameras, with AF Nikkor 35 mm f/2D lenses, were mounted on a custom-built aluminium rig fitted with two front-silvered mirrors at 45° angles, to achieve parallel view vectors matching mean human inter-pupillary separation of 63 mm^47^,^48^ (see Fig. 3c). The rig was mounted on a rotational head (Nodal Ninja with precision machined detent ring with 10° steps), allowing manual but precise rotations to capture the 18 stereo image pairs for each scene.

We initially aligned the two cameras using physical targets. We then employed MATLAB’s Camera Calibration Toolbox (www.vision.caltech.edu/bouguetj/calib_doc/) for more precise estimation of external and internal camera parameters and lens distortions, using a custom-built planar checkerboard calibration surface captured at various distances and orientations. Calibration was iterated until the angle between the camera view vectors was within 4.5 arcmin.

The focus scales marked on the two lenses were too imprecise for our purposes. To address this, the outer ring of each lens was fitted with finer, linearly spaced markings. These were verified using high contrast targets (Fig. 4d) presented at different distances. To minimize motion artefacts, the two cameras were synchronized via a wired shutter release remote control. Within the lab, synchronisation was verified using a custom-built electronic counter with 1ms resolution, which showed a maximum temporal offset of 6ms. Due to human error, a subset of stereo images in the initially captured scenes had larger temporal offsets of up to 65 m sec.

### Procedure

Aperture and exposure settings were matched for the two cameras and adapted to every scene. Four calibration images were captured at 90° azimuthal intervals. When the dynamic range of the scene was particularly high (e.g., substantial portions of an otherwise sunny scene were in the shade), the auto-bracketing feature of the cameras was used to capture 3 adjacent exposure settings. Optimal focus distance was determined by analysing the range distribution from the low resolution LiDAR scan (as in the Spheron procedure), for returns within the cameras’ vertical FOV (±12° from the horizon). Focus distance was constant across the 18 stereo pairs within each panorama.

### Colour Calibration

Colour calibration for the HDR SpheroCam and the stereo cameras was completed at the Natural Environment Research Council (NERC) Field Spectroscopy Facility, University of Edinburgh. Narrowband stimuli, incremented in 5 nm steps across the visible spectrum (390–715 nm), were presented via an integrating sphere using a double monochromator. To calibrate each camera’s response, the relative radiance of each sample was measured with a calibrated silicon photo-diode. Full details, with look up tables, are provided on the SYNS website.

### Surface Attitude Estimation

Local planar surfaces were fit to 3D neighbourhoods of points at each LiDAR point in each scene. Neighbourhood size was varied from 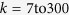 points. The position of the tangent plane was estimated as the centroid of the points, and the surface normal was identified as the eigenvector of the covariance matrix associated with the smallest eigenvalue^52^. Outlier points, defined as those deviating from the planar fit by more than three standard deviations, were removed iteratively. Degenerate elongated neighbourhoods, for which only one eigenvector is reliably estimated, were identified by examining the first two eigenvalues (*λ*_1_, *λ*_2_) and disqualifying neighbourhoods where their ratio λ_2_/λ_1_ fell below a threshold of 0.3. The smallest neighbourhood *k* for which the mean Euclidean deviation of the points from the planar fit exceeded a distance-dependent threshold was selected. For more details on the training methods used to derive these thresholds, please see the companion manuscript.

## Additional Information

**How to cite this article**: Adams, W. J. *et al*. The Southampton-York Natural Scenes (SYNS) dataset: Statistics of surface attitude. *Sci. Rep.* **6**, 35805; doi: 10.1038/srep35805 (2016).

**Publisher’s note:** Springer Nature remains neutral with regard to jurisdictional claims in published maps and institutional affiliations.

## Supplementary Material

Supplementary Information

## Figures and Tables

**Figure 1:**
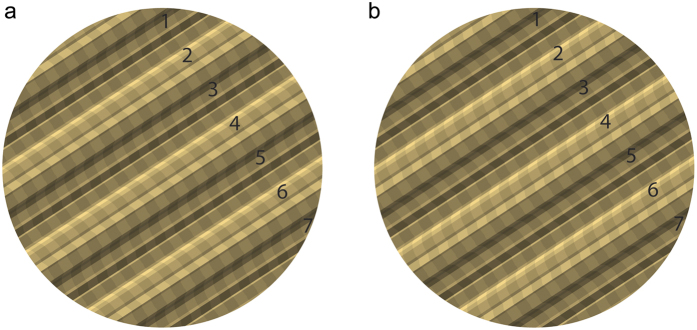
The influence of prior assumptions on perception. (**a**) Most observers perceive the even-numbered faces as convex ridges, in keeping with priors for illumination from above, and viewpoint from above. (**b**) The right image is more ambiguous – light from above dictates that even numbered ridges are convex, viewpoint from above is consistent with odd ridges being convex (see ref. 23 for similar examples).
