# Corrigendum: The Southampton-York Natural Scenes (SYNS) dataset: Statistics of surface attitude

**DOI:** 10.1038/srep42156

**Published:** 2017-03-13

**Authors:** Wendy J. Adams, James H. Elder, Erich W. Graf, Julian Leyland, Arthur J. Lugtigheid, Alexander Muryy

Scientific Reports 6: Article number: 3580510.1038/srep35805; published online: 10 26 2016; updated: 03 13 2017

A glitch in the authors’ reference-management software led to extensive errors in the reference list and in-text citations. These errors have been corrected in the PDF and HTML versions of the Article.

