# Supplementary material for: The Southampton-York Natural Scenes (SYNS) dataset: Statistics of surface attitude
Source: Sci Rep. 2016 Oct 26;6:35805. doi: 10.1038/srep35805 (PMC5080654; doi:10.1038/srep35805)
Supplement: Supplementary Information [file srep35805-s1.pdf]

The Southampton-York Natural Scenes (SYNS) dataset: Statistics of surface attitude:

Supplementary Information

Wendy J. Adams[1*], James H. Elder[2], Erich W. Graf[1], Julian Leyland[3], Arthur J. Lugtigheid[1], Alexander Muryy[1]

1. Centre for Vision and Cognition, Psychology, University of Southampton, UK
2. Centre for Vision Research, York University, Canada
3. Geography and Environment, University of Southampton, UK

*Corresponding author:

Wendy J. Adams
Psychology,
University of Southampton
Southampton, SO17 1BJ
ENGLAND

Tel: +011 (44) 0238059 3629
Email: w.adams@soton.ac.uk

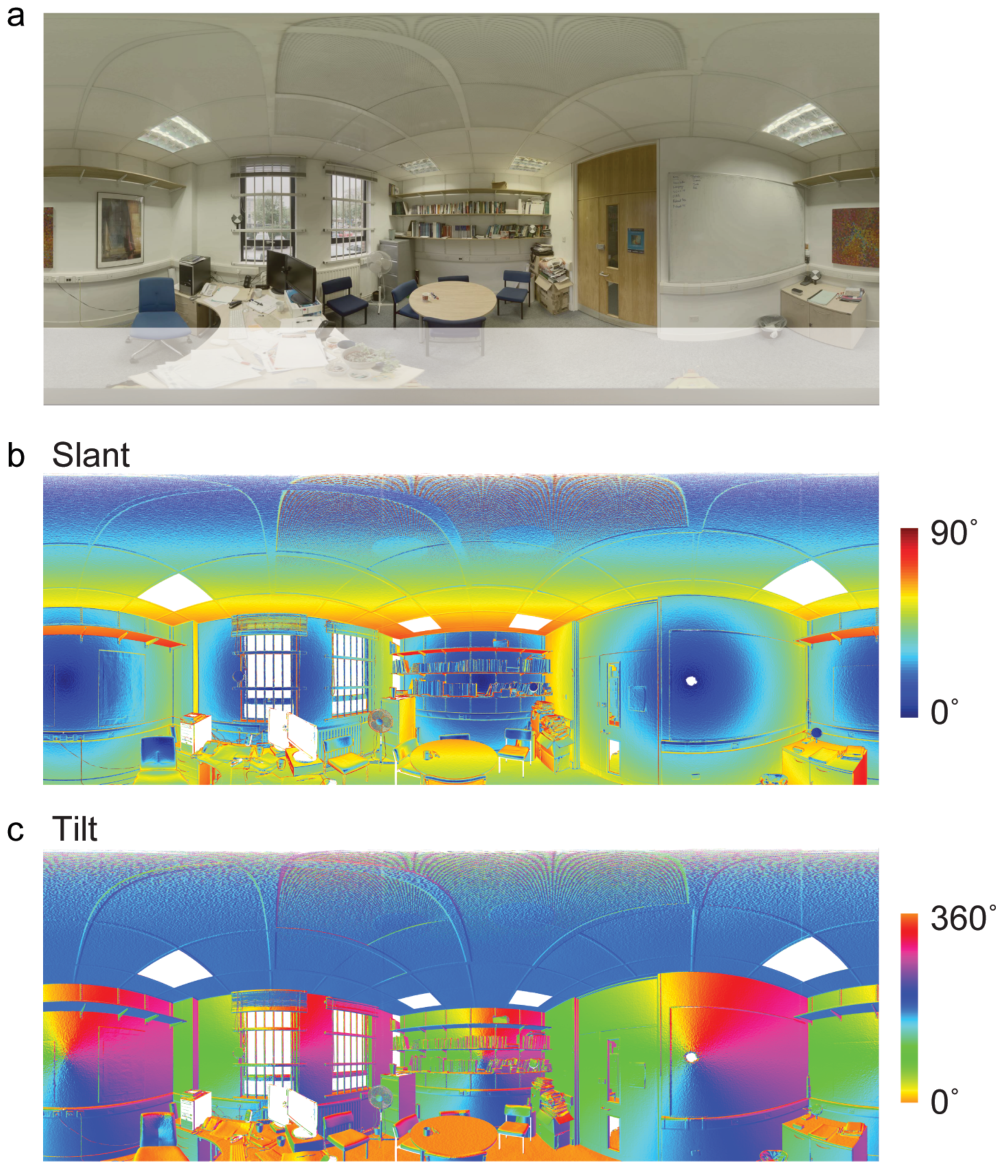

Figure S1. Surface attitude estimation for an example indoor scene. a) Spheron image; masked regions correspond to the low elevation region not captured by LiDAR. b) Egocentric slant. c) Egocentric tilt.

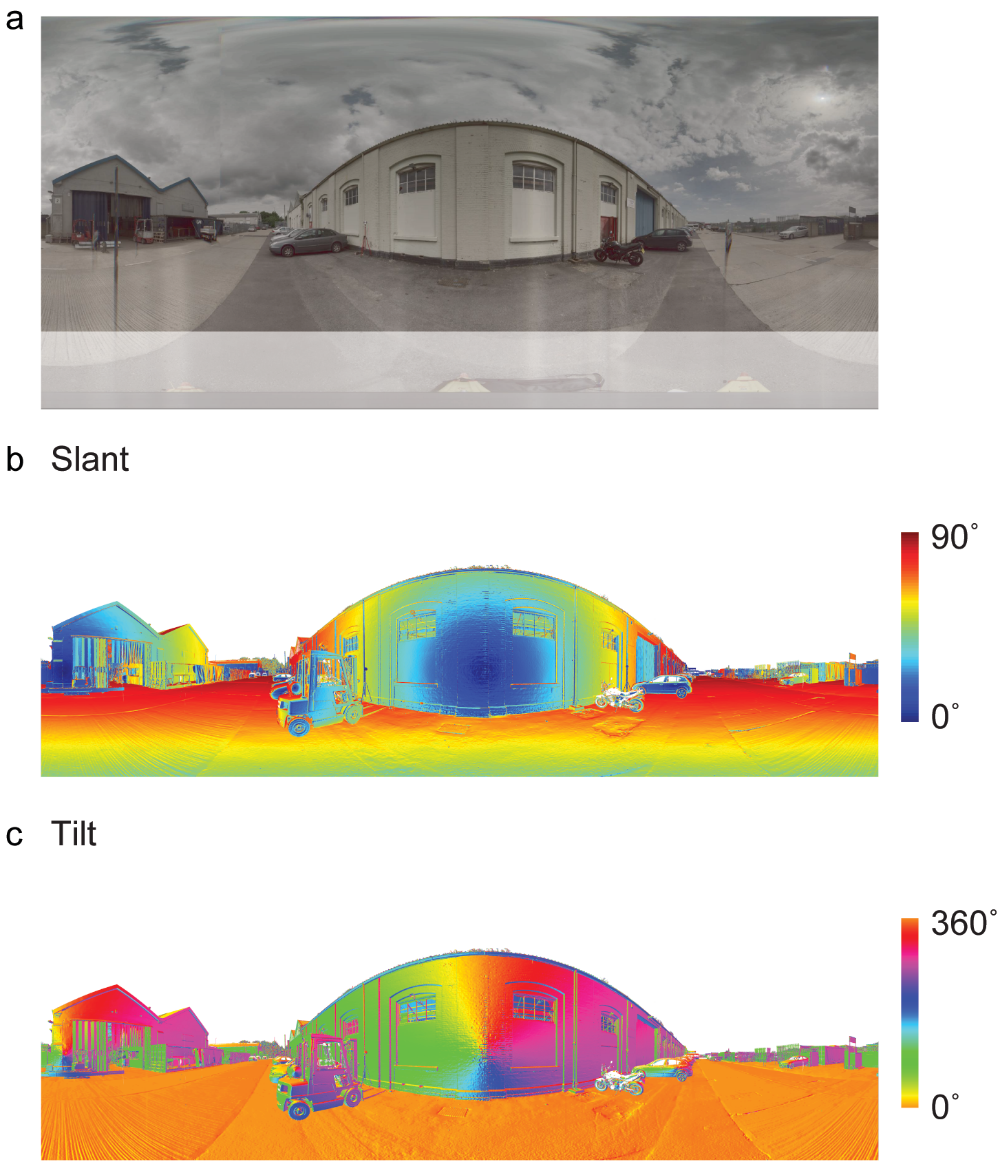

Figure S2. Surface attitude estimation for an example outdoor built scene. a) Spheron image; masked regions correspond to the low elevation region not captured by LiDAR. b) Egocentric slant. c) Egocentric tilt.

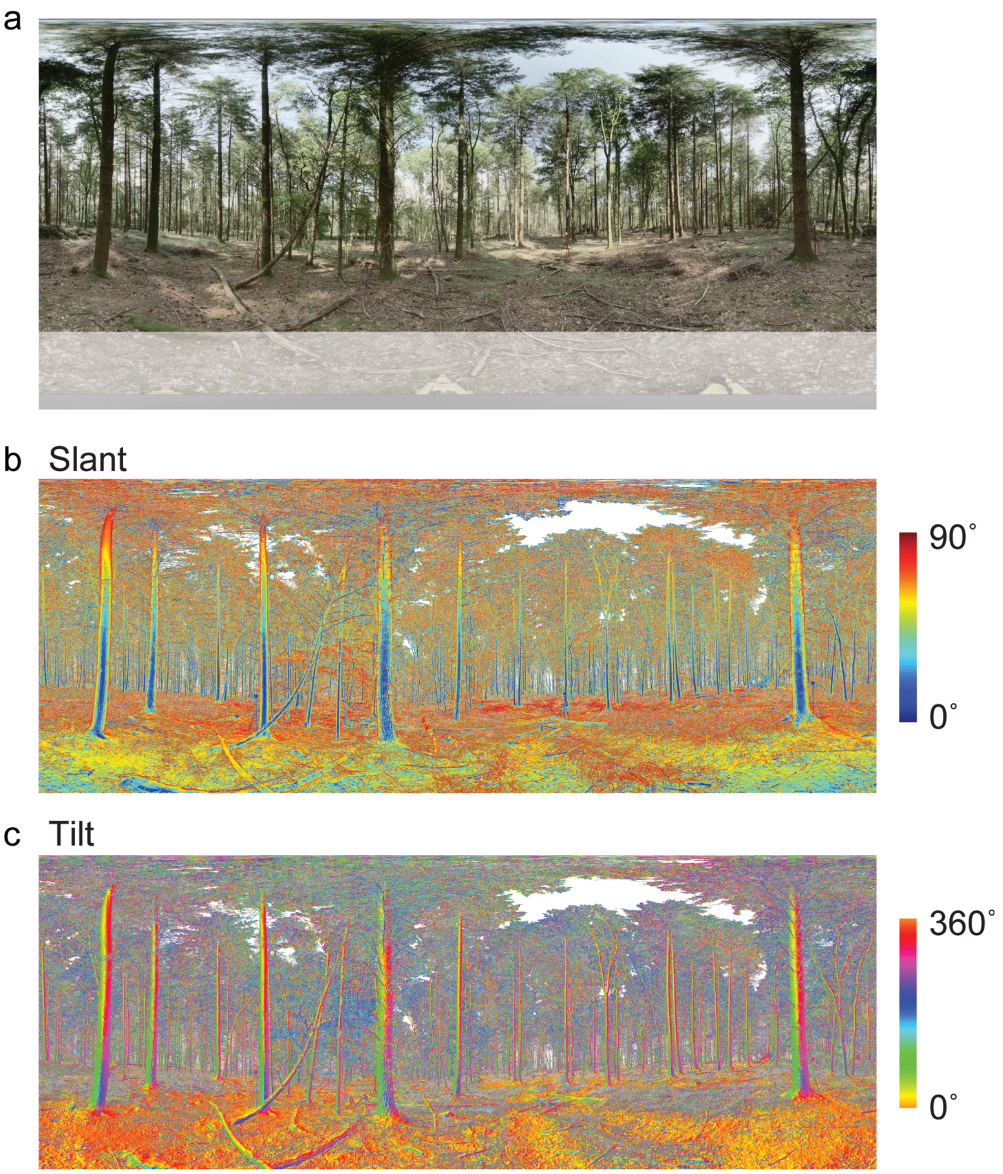

Figure S3. Surface attitude estimation for an example outdoor natural scene. a) Spheron image; masked regions correspond to the low elevation region not captured by LiDAR. b) Egocentric slant. c) Egocentric tilt.
